# Leveraging the McGeer Criteria to Estimate the Frequency of Inappropriate Antibiotic Prescribing for Urinary and Respiratory Tract Infections Relative to the Onset of the COVID-19 Pandemic at a Skilled Nursing Facility

**DOI:** 10.3390/antibiotics14010035

**Published:** 2025-01-05

**Authors:** Paulina M. Colombo, Ferris A. Ramadan, Dilsharan Kaur, Darunee Armenta, Peter P. Patterson, Katherine D. Ellingson

**Affiliations:** 1 Department of Epidemiology and Biostatistics, Mel and Enid Zuckerman College of Public Health, University of Arizona, Tucson, AZ 85724, USA; 2 Clinical Translational Sciences, University of Arizona Health Sciences, Tucson, AZ 85721, USA; 3 Patterson LTC Consults, Surprise, AZ 85374, USA

**Keywords:** antimicrobial stewardship, COVID-19, skilled nursing facility, antibiotic prescribing, McGeer criteria

## Abstract

**Background/Objectives**: The COVID-19 pandemic affected antimicrobial stewardship in healthcare, including Skilled Nursing Facilities (SNFs). This study aimed to (1) assess the appropriateness of antibiotic prescriptions for urinary tract infections (UTIs) and respiratory tract infections (RTIs) and identify predictors of inappropriate use; (2) analyze changes in prescribing practices relative to the pandemic’s onset. **Methods**: A retrospective review of electronic medical records from a 300-bed SNF (March 2019–March 2021) identified suspected UTIs and RTIs based on laboratory tests and antibiotic requests. Antibiotic prescription appropriateness was defined by clinical and microbiological alignment with the McGeer criteria, which are standardized infection definitions for long-term care residents, for UTI and RTI. Logistic regression models identified predictors of inappropriate prescribing, and an interrupted time-series analysis (ITS) examined trends relative to the pandemic onset (11 March 2020) in Arizona. **Results**: Among 370 antibiotic prescriptions, 77% of UTI and 61% of RTI prescriptions were inappropriate per the McGeer criteria. Acute dysuria and increased urgency were associated with lower odds of inappropriate UTI prescribing. For RTIs, a positive COVID-19 test increased the odds of inappropriate prescribing, while fever and acute functional decline lowered them. UTI prescriptions and inappropriateness overall increased during the pandemic, but no significant ITS trends emerged. For RTIs, no significant changes in prescribing or inappropriateness relative to the pandemic were observed. Findings emphasize the need for robust antimicrobial stewardship during and after public health emergencies.

## 1. Introduction

Long-term care (LTC) settings, including Skilled Nursing Facilities (SNF), are prone to the overuse of antibiotics due to concerns about infection spread in congregate living environments, the high susceptibility of immunosuppressed residents, and resource limitations, such as limited diagnostic tools [1]. The magnitude of antibiotic consumption in SNFs is a well-documented phenomenon, with an estimated 49% to 79% of residents prescribed at least one antibiotic during their stay [2]. In addition to high prescription rates, the majority (40–70%) of antibiotics used in these settings have been determined inappropriate or unnecessary [3]. As a result of frequent and often prolonged antibiotic usage, these settings also perpetuate the transmission of multidrug-resistant organism (MDRO) pathogens, with an estimated 35% of residents colonized [4]. The overprescribing of antibiotics in LTC settings significantly contributes to antimicrobial resistance (AMR), which has been consistently named a top global public health threat due to the increasing emergence of MDROs [5]. Inappropriate antibiotic use accelerates the development of resistant strains, making infections harder to treat and increasing the risk of disease spread, severe illness, and death [6]. The revised McGeer criteria, developed to standardize infection definitions for surveillance purposes in LTC populations, were designed specifically to address the unique clinical and microbiologic criteria in LTC residents [7]. While clinical discretion lies with the treating provider in individual cases, these criteria offer surveillance definitions to define to approximate true infections from non-infectious symptoms common in elderly populations in aggregate.

The COVID-19 pandemic hindered efforts to combat AMR, including inhibiting antimicrobial stewardship (AMS) interventions in clinical practice [8]. A 2022 report produced by the Centers for Disease Control and Prevention (CDC) highlighted significant losses in progress toward established AMS and AMR goals due to the burden of COVID-19 on the United States healthcare system [9]. Considerable reductions in AMR data collection and AMS programs, coupled with increases in antimicrobial use and subsequent MDRO infections, have been attributed to the pandemic. A 2021 meta-analysis revealed that as many as 75% of COVID-19 patients received antibiotics, with higher prescription rates among older individuals or those on mechanical ventilation [10]. This finding was supported by a 2023 systematic review, which revealed that among hospitalized individuals, antibiotics were prescribed to 78% of COVID-19 patients, regardless of condition severity [11]. These trends in prescribing persist despite the low incidence of bacterial co-infection in COVID-19 cases [12]. Amid these shifts in prescribing trends, LTC facilities faced distinct challenges.

Confronting pre-existing resource limitations, LTC faced compounded challenges during the COVID-19 pandemic. These included staffing shortages and a critical scarcity of personal protective equipment while simultaneously caring for a population highly vulnerable to the virus [13]. SNF residents faced high mortality and morbidity as a result of the pandemic, resulting in decreases in resident census counts for many facilities due to increases in case counts and a subsequent fear of contracting the virus [14]. However, despite a history of the consistent overuse of antibiotics in these settings, the existing literature presents fluctuations in prescribing behaviors throughout the pandemic’s progression [8]. Studies in LTC have described a decrease in antibiotic prescribing overall compared to pre-pandemic estimates [8,15,16]. Conversely, there were significant increases in specific antibiotic usage (e.g., azithromycin and ceftriaxone) [16,17].

Collectively, studies have not established a clear relationship between the COVID-19 pandemic and antibiotic prescribing in LTC. Our study objectives were to (1) assess the appropriateness of antibiotic prescriptions for suspected urinary (UTI) and respiratory tract infections (RTIs) using the revised McGeer criteria standardized infection definitions and identify predictors of inappropriate antibiotic use; (2) analyze changes in prescribing practices relative to the onset of the COVID-19 pandemic within electronic medical records (EMRs) from a large, Arizona-based SNF.

## 2. Results

### 2.1. Suspected Events and Demographic Characteristics

**Figure 1:**
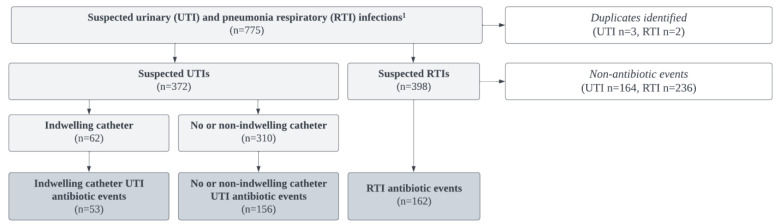
Flow diagram to demonstrate electronic medical record abstraction analysis inclusion and exclusion criteria. ^1^ A suspected infection was defined as a laboratory order (e.g., urine analysis and chest X-ray), or an antibiotic prescription.

Between March 2019 and March 2021, 775 suspected UTI and RTI infection events were abstracted; five duplicate records and any suspected infection that did not result in the prescription of an antibiotic were removed (*n* = 400). Per the McGeer criteria, UTI infections were separated by the presence or absence of an indwelling catheter or non-indwelling during urine collection. Both suprapubic and foley catheters were considered “indwelling”. The final sample consisted of 208 UTIs (indwelling *n* = 53, non-indwelling *n* = 155) and 162 RTI suspected infections that resulted antibiotic events (Figure 1).

Overall, 770 suspected UTI or RTI infections were attributed to 294 residents. On average, there were 2.6 suspected infections, and 1.5 antibiotics prescribed for each resident. Our sample consisted, on average, of older (mean = 75.6, sd = 11.3), female (62.1%, *n* = 175), and largely white (90.1%, *n* = 254) residents. A total of 18.0% (*n* = 50) of individuals were classified in the EMR as being of Hispanic origin, and most spoke English as their preferred language (93.3%, *n* = 263). Prior to their stay at this facility, the majority of residents previously resided in Pima county (Table 1).

### 2.2. Antibiotic Appropriateness and Predictors

Our kappa reliability assessment to assess the consistency of arrival at the same McGeer criteria designation (e.g., whether an antibiotic event met or did not meet the McGeer criteria for an infection) yielded an agreement rate of 96.1% (kappa statistic = 0.81). Across the study period, 77% of antibiotic prescriptions for UTIs and 61% for RTIs were deemed inappropriate.

In examining UTIs using generalized estimating equations (GEEs), those with acute dysuria had 92% lower odds of receiving inappropriate antibiotic prescriptions compared to those without acute dysuria (OR 0.08, 95% CI: 0.03–0.23). Similarly, individuals with a new or marked increase in urgency had 98% lower odds of receiving inappropriate antibiotic prescriptions compared to those without this symptom (OR 0.02, 95% CI: 0.04–0.78). Although gross hematuria was considered a potential predictor, it did not reach statistical significance (95% CI: 0.13–1.10) (Table 2).

For RTIs, the presence of fever was associated with 89% lower odds of receiving inappropriate antibiotics compared to those without fever (OR 0.11, 95% CI: 0.05–0.24). Testing positive for COVID-19 in the interval of one week prior to and one week following the suspected infection increased the odds of inappropriate prescribing for UTIs by 50% (OR 1.50, 95% CI: 1.05–2.18). Acute functional decline presented an OR (0.18) but did not meet statistical significance (Table 3).

### 2.3. Changes in Antibiotic-Prescribing Practices Relative to the Onset of the COVID-19 Pandemic

**Figure 2:**
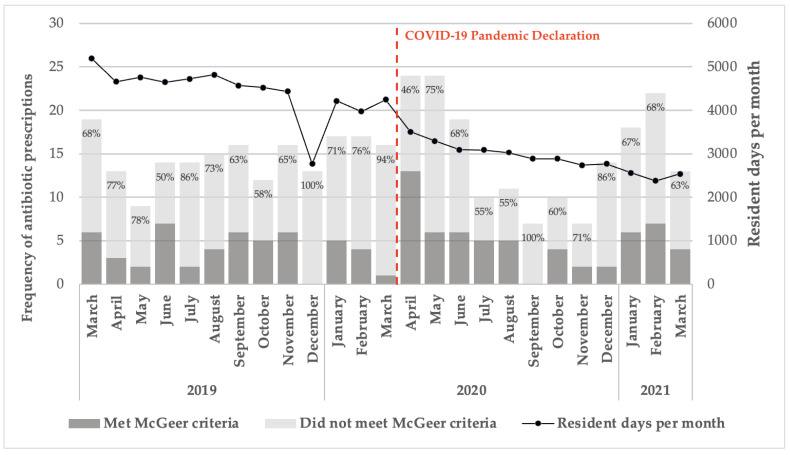
Antibiotic prescription accordance with McGeer criteria from March 2019 to 2021. Note: The frequency of antibiotic prescriptions, categorized based on adherence to McGeer criteria, is shown across the study period.

The proportion of prescriptions that did not meet the McGeer criteria fluctuated between March 2019 and March 2021. Notably, 100% of antibiotic prescriptions for suspected bacterial infections in December 2019 and 94% in March 2020 were classified as inappropriate. Resident days per month in the SNF steadily declined from approximately 5000 in March 2019 to around 3000 by March 2021 (Figure 2).

Prior to the pandemic’s onset, 60.0% of suspected UTIs in patients with indwelling catheters and 87.3% of those without catheters did not meet the McGeer criteria for appropriate antibiotic use. Similarly, 65.6% of RTI cases were classified as inappropriate for antibiotic treatment. However, there was a reduction in the rate of inappropriate antibiotic prescriptions following the pandemic onset. For UTIs in patients with indwelling catheters, there was a 13.6% decrease in observed inappropriate prescriptions. For those without catheters, the decrease was 3.4%. In the case of RTIs, there was a 10.0% reduction in inappropriate prescriptions post-pandemic declaration.

Prior to the pandemic’s onset, there was an average of 5.75 inappropriate antibiotic prescriptions for UTIs per month, which increased to 7.07 per month following the onset. Similarly, the total number of antibiotic prescriptions for UTIs rose from an average of 7.33 per month prior to the pandemic to 9.23 per month after the pandemic’s onset.

We used a generalized linear model (GLM) to evaluate the impact of the COVID-19 pandemic on prescribing practices by comparing the pooled pre-pandemic period with the pooled post-pandemic period. The model adjusted for resident days per 1000 per month to account for differences in facility occupancy over time, allowing us to assess the overall effect of the pandemic’s onset on prescribing patterns across the entire pre- and post-period. The model showed that the incidence rate of inappropriate UTI prescriptions was 3.25 times higher after the onset of the pandemic compared to before (IRR = 3.25, *p* = 0.007). For total UTI prescriptions, the incidence rate was 4.63 times higher after the pandemic began (IRR = 4.63, *p* = 0.002).

In contrast, this trend was not observed for RTIs. Before the pandemic, there were 4.75 inappropriate RTI prescriptions per month, which decreased to 3.23 per month after the pandemic onset. Similarly, total RTI prescriptions declined from 7.33 per month before the pandemic to 5.69 per month during the pandemic. GLM models for RTIs, adjusting for 1000 resident days per month, showed that these changes were not statistically significant.

### 2.4. Interrupted Time-Series Analysis

**Figure 3:**
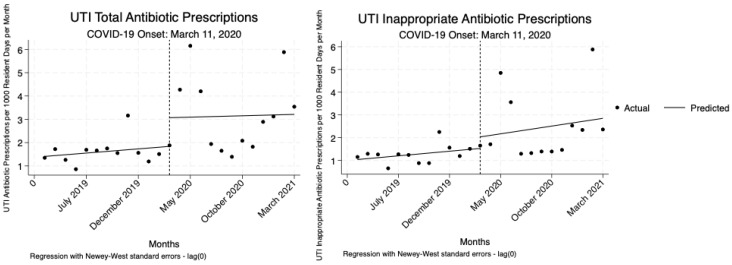
Interrupted time-series analysis to assess changes in the total and inappropriate UTI antibiotic prescribing trends before and during the COVID-19 pandemic.

The analysis indicated no statistically significant changes in total or inappropriate UTI antibiotic prescriptions per 1000 resident days per month relative to the onset COVID-19 pandemic. While there were increases observed, none of these changes reached statistical significance (Table 4, Figure 3).

**Figure 4:**
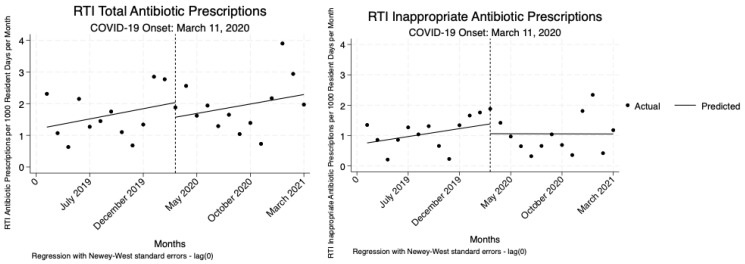
Interrupted time-series analysis to assess changes in the total and inappropriate RTI antibiotic prescribing trends before and during the COVID-19 pandemic.

For RTIs, we also observed no significant changes in either total or inappropriate antibiotic prescriptions following the onset of the COVID-19 pandemic or throughout the pandemic period. Though there were slight decreases and increases in prescribing rates, none of these shifts were statistically significant (Table 5, Figure 4).

## 3. Discussion

Using the McGeer criteria to assess the appropriateness of antibiotic prescriptions, our findings show that prescribing antibiotics consistent with standardized definitions of UTI and RTI in LTC was consistently low throughout the study period, particularly among residents with UTIs. This underscores the critical importance of standardized infection definitions in guiding antimicrobial stewardship, especially in LTC settings where the risk of inappropriate antibiotic use is high [7].

Our analysis identified acute dysuria and new or marked increase in urgency as significant predictors of appropriate antibiotic use for UTIs. These symptoms were strongly associated with adherence to the McGeer criteria, indicating their relevance in distinguishing bacterial infections that warrant antibiotic treatment. For RTIs, the presence of fever and acute functional decline were significant predictors of appropriate antibiotic prescribing. Conversely, a positive COVID-19 status emerged as a significant predictor of inappropriate antibiotic prescribing, reflecting a possible overcaution in treatment practices during the pandemic. This pattern aligns with research in nursing homes where an increase in prescribing antibiotics such as azithromycin and ceftriaxone was observed despite a lack of bacterial co-infection, often driven by COVID-19-related concerns [16].

Our observed data demonstrated a fluctuating pattern of antibiotic prescribing during the COVID-19 pandemic. This aligns with findings from Canadian LTC, where Alberta facilities experienced significant reductions in overall antibiotic prescribing rates, while Ontario facilities saw decreases in specific antibiotic classes, particularly for RTIs, without a uniform decline in overall antibiotic use [18]. Our study found that while there was a noticeable decrease in the absolute percentage of inappropriate antibiotic prescriptions for both UTIs and RTIs, the overall volume of antibiotic prescribing did not uniformly decrease. This finding is consistent with other research in LTC settings, which reports a reduction in overall antibiotic prescribing compared to pre-pandemic levels [8,15,16]. After adjusting for resident census, regression analysis revealed a significant reduction in inappropriate prescribing rates for UTIs but not for RTIs. Across both RTIs and UTIs, there were no statistically significant immediate or persistent changes in total or inappropriate antibiotic prescriptions relative to the onset of the COVID-19 pandemic. However, when we assessed the pooled pre-and post-COVID months, we found significant increases in inappropriate and total UTI prescriptions after adjusting for resident census but no significant change for RTIs. This suggests a temporary disruption in UTI prescribing driven by early pandemic uncertainty. The simpler pre/post model captured short-term disruptions, which may explain the significant increase in UTI prescriptions. Since the prescribing changes for UTIs were likely short-lived as a result of the pandemic onset, they did not persist enough to be detected in the ITS analysis.

### 3.1. Strengths

A key strength of our study is the detailed data collection within an SNF, a setting often underrepresented in research compared to acute care hospitals. The use of the McGeer criteria provided a standardized and robust framework for assessing antibiotic appropriateness, enhancing the validity of our findings. The comprehensive examination of EMRs, including clinician notes, pharmacy orders, and diagnostic reports, allowed for an in-depth analysis of each suspected infection case. The high agreement rate in data abstraction further underscores the methodological rigor and reliability of our research. Additionally, examining antibiotic prescribing patterns relative to the onset of the COVID-19 pandemic provided critical insights into the practices of antimicrobial stewardship during a global health crisis.

### 3.2. Limitations

There are a few points to consider when interpreting these findings. The study’s focus on a single SNF in Arizona may limit the generalizability of the findings to other regions or settings. The study relied on available EMR data and laboratory results, and any information not captured in these sources was not included in the analysis. We acknowledge that our assumption of absent symptoms when not documented in the EHR may not fully account for under-documentation, which could impact the completeness of our data. However, this reflects real-world data abstraction practices in retrospective studies. We also acknowledge that factors including seasonal peaks in respiratory tract infections may influence prescribing practices and warrant further investigation in future analyses. UTIs and RTIs, while common in SNFs, do not fully represent the complete scope of antibiotic prescribing practices in these settings.

The revised McGeer criteria, commonly used in infection surveillance for LTC, also come with several limitations. First, lower sensitivity for UTIs suggests the criteria may underestimate infection rates compared to clinical diagnoses [19]. Second, accurately distinguishing new infections from chronic or baseline symptoms—such as persistent cough or urinary urgency common in elderly residents—can be challenging. Third, many LTC facilities face resource limitations, with restricted access to diagnostics like radiology or laboratory testing, which can limit the accurate application of the criteria. These criteria are not intended as clinical prescribing guidelines for benchmarking prescribing practices. In this paper, we have used these definitions to estimate “inappropriate prescribing”.

Further research is warranted to explore the long-term impacts of the COVID-19 pandemic on antimicrobial prescribing patterns across various healthcare settings. The findings from this study can inform the development of more resilient healthcare policies, especially in enhancing AMS programs in LTC facilities.

Our study reveals largely consistent inappropriate prescribing patterns for antibiotics within an SNF during the COVID-19 pandemic. These patterns underscore the challenges faced by healthcare providers in maintaining appropriate antibiotic use amidst the pandemic despite emphasis on AMS programs in the pre-pandemic period [20,21], These findings highlight the need for effective AMS interventions that are sustainable during public health emergencies.

## 4. Materials and Methods

### 4.1. Study Design and Setting

In this retrospective cohort study, EMRs from a cohort of SNF residents with suspected UTI or RTI were systematically reviewed for the period March 2019 to March 2021. The study site was a 300-bed Arizona SNF with an AMS program in place.

### 4.2. Study Sample

Data were abstracted from the SNF’s EMR system between the study period of March 2019 to March 2021 for suspected case (s) of UTIs or RTIs that resulted in an antibiotic prescription. Suspected UTIs and RTIs, defined by either healthcare provider requests for laboratory testing (e.g., a chest X-ray and/or urinary analysis) or the initiation of antibiotic therapy, were flagged within the facility EMR. Resident data were excluded if (1) suspected infections were duplicate records for the same suspect infection occurring within one week of the index suspect infection event for the same resident (in these instances, the first record was used), (2) suspected infections did not result in an antibiotic prescription, or (3) the resident had an admittance period shorter than one day (Figure 1).

### 4.3. Electronic Medical Record Abstraction

We designed our data abstraction protocol based on the elements needed to apply the McGeer criteria, a standardized set of infection definitions employed for surveillance purposes within LTC settings [7] for UTIs (both indwelling and non-indwelling) and pneumonia-associated RTIs. We selected the revised McGeer Criteria over the Loeb Minimum Criteria [22] due to their more recent update and enhanced diagnostic specificity, particularly in retrospective analyses. The revised McGeer Criteria provide stricter thresholds, including microbiologic confirmation, which we found critical for evaluating antibiotic appropriateness during the COVID-19 pandemic. Following suspected infection identification, we used a Research Electronic Data Capture (REDCap) EMR abstraction tool to extract McGeer components for each suspected infection type [23,24]. Three abstractors were assigned a random selection of EMRs. They each conducted a thorough review of clinician progress notes, microbiology information from laboratory reports, pharmacy orders, and vital statistics to gather comprehensive data on each suspected infection. Patient demographic characteristics were also collected.

EMR documents were reviewed during the time frame extending one week prior to and one following the date of either a laboratory report or antibiotic prescription—whichever occurred first. This included laboratory reports sourced from the SNF’s diagnostic testing facility, including bacterial counts in urine culture reports and chest X-ray results. In instances where more than one antibiotic or multiple types of laboratory testing (e.g., both urine culture and chest X-ray) were ordered within one week prior to and one week following a suspected infection, we created separate records to accurately reflect each distinct suspected infection event. For suspected RTIs, we also incorporated data from influenza and COVID-19 tests (following 11 March 2020, the COVID-19 onset-period) and influenza tests. In cases where symptoms or clinical components were not explicitly mentioned in the EHR, we assumed they were absent.

### 4.4. Statistical Analyses

#### 4.4.1. Variables

Resident EMR data from the REDCap database was used to create a binary outcome variable based on the McGeer criteria. This variable consisted of both clinical and microbiological information, including colony forming units for UTIs (see McGeer, LTC-specific infection criteria in Supplementary Materials). For the purposes of our analyses, the outcome variable was the appropriateness of an antibiotic prescription. A prescription was deemed “appropriate” if the abstracted data for a suspected infection met the McGeer criteria and “inappropriate” if the infection-specific criteria were not met. The infection types, as categorized in the criteria, included UTIs with or without indwelling catheters and RTIs. Individual covariates were created to identify the presence or absence of each McGeer-defined clinical symptom (e.g., fever, acute dysuria, cough, etc.) and microbiological criteria (e.g., the presence of an ESBL, positive COVID-19, or influenza test). In total, 14 and 12 individual clinical and symptom criteria variables were created for UTI and RTIs, respectively.

A binary variable was also generated to identify the onset of the COVID-19 pandemic. A binary variable was created to identify trends relative to the onset of the COVID-19 pandemic in Arizona. The period before the pandemic’s onset includes events from 1 March 2019 to 10 March 2020, while the period following the onset spans from 11 March 2020 to 31 March 2021, based on the World Health Organization’s declaration of the pandemic on 11 March 2020. To account for changes in the SNF census over the study period, a variable consisting of the number of days per month residents remained in the facility was created using internal facility reports. Residents with an admittance period shorter than one day were excluded from the census. A variable consisting of study time in months (1–25) was also created.

#### 4.4.2. Analyses

We assessed the consistency of arrival at the same McGeer Criteria designation for suspected infections with antibiotic use (e.g., whether suspected infection met or did not meet the McGeer criteria for an infection) across abstractors and any remaining discrepancies were resolved by the principal investigator. A kappa statistic was calculated to assess the level of agreement beyond chance.

To evaluate the appropriateness of antibiotic prescriptions from 1 March 2019 to 31 March 2021, we calculated the frequency and proportion of suspected infection events leading to antibiotic prescriptions. We then evaluated the binary outcome of antibiotic prescription appropriateness for each suspected infection, as defined by the McGeer criteria. We included all cases of suspected UTIs (e.g., cultured with no catheter, non-indwelling catheter, or indwelling catheter). We then analyzed key predictors of inappropriate antibiotic prescriptions. To achieve this, we applied GEE to independently model the appropriateness of antibiotic prescriptions in relation to each specific clinical symptoms outlined in the criteria for UTIs (*n* = 14) (Table 2) and RTIs (*n* = 11) (Table 3) separately. GEE was chosen to account for non-independence in the data as individual residents could appear multiple times in the datasets if they had multiple suspected infections. To address this, a resident identification number was included as a clustering variable, ensuring that repeated measures for the same individual were appropriately accounted for within the analysis. Given that the exposure (symptoms) and outcome (the appropriateness of prescribing) were assessed simultaneously, we used a logistic regression framework with GEE to estimate Odds Ratios (ORs) for the association between individual symptoms and inappropriate antibiotic prescribing.

Our second objective assessed changes in antibiotic prescribing practices relative to the onset of the pandemic. We conducted an ITS to determine whether prescribing patterns differed following the onset of the COVID-19 pandemic in Arizona [25]. Two separate models were run for UTIs and RTIs. The first of which modeled the number of inappropriate antibiotic prescriptions, and the second modeled the total number of antibiotic prescriptions (appropriate and inappropriate). The outcome was the number of inappropriate or total prescriptions per 1000 resident days, and the onset of the pandemic was March 2020. Study time in months (1–25) was used to set the data. The equation representing this model is shown below [26].

$$Log\left(\mu_{t}\right)=\beta_{0\;}+\beta_{1\;}T\;\left({time}_{t\;}\right)+\beta_{2\;}I_{t\;}\;{(pandemic}_{t})\;+\beta_{3\;}{TSI}_{t\;}\;\left({time}_{t\;}\times {pandemic}_{t}\right)+\beta_{4\;\;}(T_{t}+{TSI}_{t\;})$$

In this equation modeling monthly rates of antibiotic prescribing outcomes, T represents the trend before the pandemic onset, I reflects the immediate impact of the pandemic’s onset, TSI captures the difference between the post-pandemic and pre-pandemic trends, and T + TSI represents the overall post-pandemic trend.

All data analyses were performed using Stata 18 software, including the use of the “itsa” package for ITS analyses. Grammarly was employed to enhance the clarity and conciseness of the writing, and ChatGPT was infrequently used to refine the word choice during manuscript preparation. All AI-assisted suggestions were thoroughly reviewed by the authors to ensure scientific accuracy.

## Figures and Tables

**Table 1:** Demographic characteristics of residents comprising suspected infections receiving an antibiotic prescribing within an Arizona-based Skilled Nursing Facility between March 2019 and 021, *n* = 163 ^1^.

	Mean	*Sd*	*Range*
Age	75.9	11.5	37.0–103.0
Resident Census (days per month)	3854.5	864.9	2382.0–5197.0
Suspected Infections per Resident	2.6	2.6	1–17
Antibiotic Prescriptions per Resident	2.0	1.0	0–13
	*n* (%)		
Sex			
*Male*	64 (39.3)		
*Female*	99 (60.7)		
Race			
White	145 (89.0)		
Black or African American	8 (4.9)		
American Indian/Alaska Native	3 (1.8)		
Unknown	7 (4.3)		
Ethnicity			
Not of Hispanic origin	128 (78.5)		
Hispanic	29 (17.8)		
Unknown	6 (3.7)		
*Preferred language*			
*English*	152 (93.3)		
*Spanish*	11 (6.8)		
*County previously resided in*			
*Pima*	271 (92.2)		
*Other*	23 (7.8)		

^1^ A total of 370 non-duplicate antibiotic events for suspected UTI and RTIs were attributed to 163 residents.

**Table 2:** Unadjusted generalized estimating equations (GEEs) to model predictors of inappropriate antibiotic prescribing for urinary tract infections (UTIs) without an indwelling catheter ^1^ between March 2019 and 2021 (*n* = 156).

	UTI			
	% Inappropriate	% Appropriate	OR (95% CI)	p
Acute dysuria	9.8	60.9	0.08 (0.03–0.23)	**<0.0001**
Acute pain, swelling, or tenderness of the testes, epididymis, or prostate	1.5	4.4	0.27 (0.02–3.16)	0.30
Fever or leukocytosis	49.0	43.5	1.57 (0.91–2.71)	0.11
Acute costovertebral angle pain or tenderness	omitted due to small sample sizes			
Suprapubic pain	omitted due to small sample size			
Gross hematuria	3.8	26.1	0.38 (0.13–1.10)	0.08
New or marked increase of incontinence	12.0	21.7	0.92 (0.33–2.55)	0.87
New or marked increase of urgency	0.8	8.7	0.18 (0.04–0.78)	0.02
New or marked increase of frequency	0.8	13.0	0.43 (0.09–2.13)	0.304
Acute change in mental status from baseline	8.3	13.0	1.31 (0.32–5.36)	0.70
Acute functional decline	9.0	8.7	0.72 (0.26–2.09)	0.54
Foul-smelling urine	11.3	13.0	0.97 (0.39–2.42)	0.95
Dark urine	20.3	26.1	1.80 (0.84–3.85)	0.13
Presence of ESBL	24	8.7	1.52 (0.45–5.19)	0.50

^1^ Suspected infections for patients with an indwelling catheter were excluded due to small sample sizes.

**Table 3:** Unadjusted generalized estimating equations (GEEs) to model predictors of inappropriate antibiotic prescribing for respiratory tract infections (RTIs) between March 2019 and 2021 (*n* = 162).

	RTI			
	% Inappropriate	% Appropriate	OR (95% CI)	*p*
New or increased cough	58.6	52.4	1.15 (0.64–2.09)	0.64
New or increased sputum production	30.3	19.1	1.36 (0.69–2.70)	0.38
O_2_ saturation < 94% on room air or a reduction in O_2_ saturation of >3% from baseline	77.8	84.1	0.70 (0.32–1.54)	0.38
New or changed lung examination abnormalities	61.6	65.1	0.91 (0.48–1.70)	0.76
Pleuritic chest pain	2.0	1.6	0.80 (0.11–6.15)	0.83
Respiratory rate of >= 25 breaths/min	2.0	6.4	0.50 (0.11–2.33)	0.38
Fever	44.4	90.5	0.11 (0.05–0.24)	<0.01
Leukocytosis	15.2	28.6	0.55 (0.26–1.14)	0.11
Acute change in mental status from baseline	2.0	4.8	0.59 (0.11–3.15)	0.54
Acute functional decline	2.0	9.5	0.18 (0.03–0.99)	0.05
COVID-19 positive test ^1^	37.5	16.7	1.65 (1.07–2.56)	0.02
Influenza	0	9.1	1.40 (0.88–2.24)	0.16

^1^ The COVID-19-positive test model included only antibiotic prescriptions for suspected RTIs following the onset of the COVID-19 pandemic using the World Health Organization pandemic declaration (11 March 2020).

**Table 4:** Interrupted time-series analysis using generalized linear models (GLMs) to model the impact of COVID-19 on inappropriate and total antibiotic prescribing practices for UTI between March 2019 and 2021.

			Total UTI Antibiotic Prescriptions *			Inappropriate UTI Antibiotic Prescriptions **		
**Parameter**	**Variable (s)**	Coefficient (s)	IRR (95% CI)	% Change	*p*	IRR (95% CI)	% Change	*p*
Intercept	Constant	$\beta_{0\;}$	*…*	*…*	*…*	*…*	*…*	*…*
Pre-COVID trend	T	$\beta_{1\;}$	1.04 (0.95–1.13)	3.7	0.39	1.04 (0.99–1.10)	4.2	0.11
Immediate COVID impact	I	$\beta_{2}$	3.42 (0.40–29.13)	242.2	0.25	1.66 (0.31–8.99)	66.1	0.54
Change during COVID trend	TSI	$\beta_{3\;}$	0.98 (0.74–1.29)	−2.4	0.86	1.03 (0.853–1.269)	2.8	0.83
Persistence of trend during COVID	T + TSI	$\beta_{1}+\beta_{3\;}$	1.01 (0.78–1.32)	1.2	0.93	1.07 (0.925–1.309)	7.0	0.58

Note: In this equation modeling monthly rates of antibiotic prescribing outcomes, T represents the trend before the pandemic onset, I reflects the immediate impact of the pandemic’s onset, TSI captures the difference between the post-pandemic and pre-pandemic trends, and T + TSI represents the overall post-pandemic trend. * Modeling the total number of antibiotics (both inappropriate and appropriate) prescribed for suspected UTIs per 1000 resident facility days per month, COVID-19 pandemic, study time by month, and an interaction term between two time-varying covariates: study time by month and pandemic variable (pre COVID-19: 1 March 2019–11 March 2020, during COVID-19: 11 March 2020–31 March 2021). ** Modeling the number of inappropriate antibiotic prescriptions for suspected UTIs per 1000 resident facility days per month, COVID-19 pandemic, study time by month, and an interaction term between two time-varying covariates: study time by month and pandemic variable (pre COVID-19: 1 March 2019–11 March 2020, during COVID-19: 11 March 2020–31 March 2021).

**Table 5:** Interrupted time-series analysis using generalized linear models (GLMs) to understand the impact of COVID-19 on inappropriate and total antibiotic prescribing practices for RTIs between March 2019 and 2021.

			Total RTI Antibiotic Prescriptions *			Inappropriate RTI Antibiotic Prescriptions **		
**Parameter**	**Variable (s)**	Coefficient (s)	IRR (95% CI)	% Change	*p*	IRR (95% CI)	% Change	*p*
Intercept	Constant	$\beta_{0\;}$	*…*	*…*	*…*	*…*	*…*	*…*
Pre-COVID trend	T	$\beta_{1\;}$	1.07 (0.91–1.24)	6.7	0.39	1.05 (0.96–1.15)	5.4	0.23
Immediate COVID impact	I	$\beta_{2}$	0.63 (0.17–2.34)	−37.1	0.47	0.75 (0.28–1.85)	−27.6	0.48
Change during-COVID trend	TSI	$\beta_{3\;}$	1.00 (0.82–1.21)	−0.5	0.96	0.95 (0.82–1.09)	−5.1	0.44
Persistence of trend during-COVID	T + TSI	$\beta_{1}+\beta_{3\;}$	1.06 (0.94–1.20)	6.2	0.33	1.00 (0.90–1.11)	−0.1	0.99

Note: In this equation modeling monthly rates of antibiotic prescribing outcomes, T represents the trend before the pandemic onset, I reflects the immediate impact of the pandemic’s onset, TSI captures the difference between the post-pandemic and pre-pandemic trends, and T + TSI represents the overall post-pandemic trend. * Modeling the total number of antibiotics (both inappropriate and appropriate) prescribed for suspected RTIs per 1000 resident facility days per month, COVID-19 pandemic, study time by month, and an interaction term between two time-varying covariates: study time by month and pandemic variable (pre COVID-19: 1 March 2019–11 March 2020, during COVID-19: 11 March 2020–31 March 2021). ** Modeling the number of inappropriate antibiotic prescriptions for suspected RTIs per 1000 resident facility days per month, COVID-19 pandemic, study time by month, and an interaction term between two time-varying covariates: study time by month and pandemic variable (pre COVID-19: 1 March 2019–11 March 2020, during COVID-19: 11 March 2020–31 March 2021).

## Data Availability

Detailed clinical data are not available. Requests for de-identified data can be sent to the corresponding author, Dr. Katherine Ellingson.

## Supplementary Materials

The following supporting information can be downloaded at https://www.mdpi.com/article/10.3390/antibiotics14010035/s1, Table S1. Urinary Tract Infection (UTI) Surveillance Definitions. Table S2. Respiratory Tract Infection (UTI) Surveillance Definitions. Table S3. Infection Constitutional Criteria.
