# Supplementary material for: Leveraging the McGeer Criteria to Estimate the Frequency of Inappropriate Antibiotic Prescribing for Urinary and Respiratory Tract Infections Relative to the Onset of the COVID-19 Pandemic at a Skilled Nursing Facility
Source: Antibiotics (Basel). 2025 Jan 5;14(1):35. doi: 10.3390/antibiotics14010035 (PMC11759821; doi:10.3390/antibiotics14010035)
Supplement: Supplementary file 1 [file antibiotics-14-00035-s001.zip › antibiotics-3346173-supplementary.pdf]

<u>**Supplementary Information**</u>

Supplementary S1

Supplementary Material Table S1. Urinary Tract Infection (UTI) Surveillance Definitions

**UTI** (without indwelling catheter)[1]	**UTI** (with indwelling catheter)[2]
Must fulfill both 1 AND 2.<br>**1. At least one of the following sign or symptoms**<br>- Acute dysuria or pain, swelling, or tenderness of testes, epididymis, or prostate<br>- Fever or leukocytosis **AND** at least one of the following: acute costovertebral angle pain or tenderness, suprapubic pain, gross hematuria, new or marked increase in incontinence, new or marked increase in urgency, or new or marked increase in frequency<br>- If no fever or leukocytosis, then ≥ 2 of the following: suprapubic pain, gross hematuria, new or marked increase in incontinence, new or marked increase in urgency, or new or marked increase in frequency<br>**2. At least one of the following microbiologic criteria**<br>- $\geq 10^5$ cfu/mL of no more than 2 species of organisms in a voided urine sample<br>- $\geq 10^2$ cfu/mL of any organism(s) in a specimen collected by an in-and-out catheter	*Must fulfill both 1 AND 2.*<br>**1. At least one of the following sign or symptoms**<br>- Fever, rigors, or new-onset hypotension, with no alternate site of infection<br>- Either acute change in mental status or acute functional decline, with no alternate diagnosis and leukocytosis<br>- New-onset suprapubic pain or costovertebral angle pain or tenderness<br>- Purulent discharge from around the catheter or acute pain, swelling, or tenderness of the testes, epididymis, or prostate<br>**2. Urinary catheter specimen culture with $\geq 10^5$ cfu/mL of any organism(s)**

[1] *UTI can be diagnosed without localizing symptoms if a blood isolate is the same as the organism isolated from urine and there is no alternate site of infection.*

[2] *Recent catheter trauma, catheter obstruction, or new onset hematuria are useful localizing signs that are consistent with UTI but are not necessary for diagnosis.*

Supplementary Material Table S2. Respiratory Tract Infection (UTI) Surveillance Definitions

**Pneumonia**[1]
Must fulfill 1, 2, AND 3.<br>**1. Chest X-ray with pneumonia or a new infiltrate**<br>**2. At least one of the following criteria**<br>- New or increased cough<br>- New or increased sputum production<br>- O2 sat <94% on room air, or >3% decrease from baseline O2 sat<br>- New or changed lung exam abnormalities<br>- Pleuritic chest pain<br>- Respiratory rate ≥ 25 breaths/min<br>**3. At least one of the following criteria**<br>- Fever<br>- Leukocytosis<br>- Acute mental status change<br>- Acute functional decline

[1] *Conditions mimicking the presentation of RTI (e.g., congestive heart failure or interstitial lung diseases) should be excluded.*

Supplementary S2

Supplementary Material Table S3. Infection Constitutional Criteria

Fever	Leukocytosis	Acute Mental Status Change	Acute Functional Decline
Single oral temp >37.8 ^{0}C (100 ^{0}F),<br>**OR**<br>Repeated oral temp >37.2 ^{0}C (99 ^{0}F),<br>**OR**<br>Repeated rectal temp >37.5 ^{0}C (99.5 ^{0}F),<br>**OR**<br>Single temp >1.1 ^{0}C (2 ^{0}F) from baseline from any site	>14,000 WBC/mm3,<br>**OR**<br>>6% band,<br>**OR**<br>≥1,500 bands/mm3	Acute onset,<br>**AND**<br>Fluctuating course,<br>**AND**<br>Inattention,<br>**AND**<br>Either disorganized thinking, **OR** altered level of consciousness	**3-point increase in baseline ADL score according to the following items**<br>1. Bed mobility<br>2. Transfer<br>3. Locomotion within LTC<br>4. Dressing<br>5. Toilet use<br>6. Personal hygiene<br>7. Eating<br>[Each scored from 0 (independent) to 4 (total dependence)]
